# News and Coming Events

**Published:** 1908-10-10

**Authors:** 


### NEWS AND COMING EVENTS.

Dr. G. T. D. Cooper has been promoted medical officer of the General Hospital, Penang, Straits Settlements.

The Salters' Company have voted £100 per annum for a period of three years to the cancer research laboratories of the Middlesex Hospital as a research scholarship.

His Majesty has been pleased to grant permission to Dr. Vincent Dickinson to accept and wear the Order of Chevalier of the Crown of Italy conferred on him by the King of Italy in recognition of his services as physician to the Italian Hospital.

On September 24 Sir Charles Dilke, M.P., in the presence of a large gathering formally opened the new hospital for Lydney and district. The building comprises two wards of four beds each, operation theatre, dispensary, etc. The site was given by Mr. C. Bathurst, and Mr. R. Thomas has defrayed the whole cost of building the hospital.

On October 1 the new food regulations which have been drawn up by the Local Government Board in pursuance of the Public Health Act, 1907, came into force. The regulations are framed with the object of insuring that articles of food which are unfit for human consumption shall be dealt with at the port of discharge, so that the danger to the public health from the consumption of unwholesome food may be prevented. The medical officer of health is empowered by the regulations to examine any articles of food landed within his district, and if, in his opinion, an article is unwholesome, he may prevent its sale until it has been examined by a magistrate. If the magistrate is satisfied that the article he examines is unsound he is required to condemn the food and order its destruction, unless it is proved to him that the article is not intended for sale for human consumption.

The fourth annual Medical Exhibition organised by the *British and Colonial Druggist* was opened on Monday, October 5, at the Horticultural Hall, and remained open until Friday, October 9. This exhibition was not intended as a place of entertainment for the general public, to whom was charged a high admission fee, but as a means of enabling members of the medical profession and persons connected with hospital management to note the latest improvements in medical and surgical appliances, drugs, invalid foods, and so forth. More than one hundred firms were represented. The Exhibition was well arranged and of considerable interest. There was a reception room for professional men attending, and in addition to many comforts and attractions specially prepared for visitors an orchestra played throughout the day.

The League of Mercy is now drawing to the close of its tenth year's work. Founded in 1899 by the King, when Prince of Wales, as an auxiliary to the Prince of Wales's (now King Edward's) Hospital Fund for London, its membership is steadily increasing every year. To December 31 last it had contributed £97,000 to London hospitals through the King's Fund and made grants of nearly £2,000 to hospitals outside the radius of the fund in country districts where its work is supported. The aim of the League, besides augmenting the resources of hospitals, is to form a complete organisation whereby may be enlisted the personal services of those who are willing to assist in succouring the sick and the afflicted. In the League, which has now forty-seven London and fifty-nine country districts, the poor have a channel through which they may make some return for the benefits they receive from London hospitals and those of the home counties.

On Monday, October 19, and five following days, the recently completed buildings of the new Manchester Royal Infirmary will be opened for public inspection.

The Emperor of Japan has sent a donation of £500 to the Seamen's Hospital Society, in recognition of the assistance rendered to Japanese subjects by the *Dreadnought* Hospital at Greenwich.

On Tuesday, October 20, at 8 p.m., in connection with the Royal Dental Hospital of London, there will be held a *conversazione* and distribution of prizes by Lord Alverstone at the Royal Institute Galleries, Prince's Hall, Piccadilly.

The opening of the winter course of lectures at the Central London Throat and Ear Hospital, Gray's Inn Road, W.C., will take place on Friday, October 16, at 3 p.m., on which occasion Dr. John Macintyre, F.R.S.E.,, Glasgow Royal Infirmary, will deliver an address on "The Recent Methods of Examination of the Nose and Throat," illustrated by lantern slides and instruments.

The quarterly meeting of the Board of Delegates of the Hospital Saturday Fund was held at the offices, Gray's Inn Road, on October 3, Sir Savile Crossley (Chairman) presiding. The report of the Finance Committee showed that from January 7 to September 19, 1908, the general collection amounted to £12,622, which with local collections and other receipts made a total of £13,531, as against £12,602 in the corresponding period of 1907. Deducting payments, there remained a balance of £11,908. Adding the balance of the previous year the total balance was £12,502. The total balance for the previous year was £11,504. The committee have placed £12,000 on deposit at Messrs. Hoare and Co.'s Bank. October 17 has been fixed for Hospital Saturday, and the annual dinner of the fund will be held on February 13.

The Harveian Oration before the Royal College of Physicians of London will be delivered by Dr. J. A. Ormerod, physician to St. Bartholomew's Hospital, London, on Monday, October 19, at 4 p.m., and in the evening the customary Harveian dinner will take place.

At Haslar Hospital on September 29, Dr. T. J. Macnamara, M.P., Secretary to the Admiralty, presented prizes to the medical officers just entering the naval service. Inspector-General G. D. Gimlette, C.B., presided at the ceremony. Dr. Gimlette said he was able to give a very good account of the medical officers who were passing into the Fleet. He read the report of Fleet Surgeon Bassett-Smith, which referred to the changes that have been made in the instructional course for surgeons in the Navy, the matters which have received additional attention being the examination of recruits, the management and treatment of diseases to which seamen are prone, and the administration of anæsthetics. Dr. Macnamara, addressing the surgeons, said he wished them and the medical schools generally to understand that, so far as the Admiralty was concerned, the department meant to see that the naval hospitals were well found, and their doctors as good as any in their great profession. The doctors, on their part, were bound to help the Admiralty; they were bound to be ever up to date, keen, and enthusiastic in their calling. They entered a field of practice second to none in width, variety, and interest. They must not only be ready to apply the latest knowledge to the cure of the ailing; they must also be alert with the most scientific measures for the prevention of disease.

